# Plan Quality and Treatment Efficiency for Radiosurgery to Multiple Brain Metastases: Non-Coplanar RapidArc vs. Gamma Knife

**DOI:** 10.3389/fonc.2016.00026

**Published:** 2016-02-11

**Authors:** Haisong Liu, David W. Andrews, James J. Evans, Maria Werner-Wasik, Yan Yu, Adam Paul Dicker, Wenyin Shi

**Affiliations:** ^1^Department of Radiation Oncology, Thomas Jefferson University, Philadelphia, PA, USA; ^2^Department of Neurological Surgery, Thomas Jefferson University, Philadelphia, PA, USA

**Keywords:** VMAT, RapidArc, Gamma Knife, SRS, brain metastasis

## Abstract

**Objectives:**

This study compares the dosimetry and efficiency of two modern radiosurgery [stereotactic radiosurgery (SRS)] modalities for multiple brain metastases [Gamma Knife (GK) and LINAC-based RapidArc/volumetric modulated arc therapy], with a special focus on the comparison of low-dose spread.

**Methods:**

Six patients with three or four small brain metastases were used in this study. The size of targets varied from 0.1 to 10.5 cc. SRS doses were prescribed according to the size of lesions. SRS plans were made using both Gamma Knife^®^ Perfexion and a single-isocenter, multiple non-coplanar RapidArc^®^. Dosimetric parameters analyzed included RTOG conformity index (CI), gradient index (GI), 12 Gy isodose volume (*V*_12Gy_) for each target, and the dose “spread” (Dspread) for each plan. Dspread reflects SRS plan’s capability of confining radiation to within the local vicinity of the lesion and to not spread out to the surrounding normal brain tissues. Each plan has a dose (Dspread), such that once dose decreases below Dspread (on total tissue dose–volume histogram), isodose volume starts increasing dramatically. Dspread is defined as that dose when volume increase first exceeds 20 cc/0.1 Gy dose decrease.

**Results:**

RapidArc SRS has smaller CI (1.19 ± 0.14 vs. 1.50 ± 0.16, *p* < 0.001) and larger GI (4.77 ± 1.49 vs. 3.65 ± 0.98, *p* < 0.01). *V*_12Gy_ results were comparable (2.73 ± 1.38 vs. 3.06 ± 2.20 cc, *p* = 0.58). Moderate to lower dose spread, V6, V4.5, and V3, were also equivalent. GK plans achieved better very low-dose spread (≤3 Gy) and also had slightly smaller Dspread, 1.9 vs. 2.5 Gy. Total treatment time for GK is estimated between 60 and 100 min. GK treatments are between 3 and 5 times longer compared to RapidArc treatment techniques.

**Conclusion:**

Dosimetric parameters reflecting prescription dose conformality (CI), dose fall off (GI), radiation necrosis indicator (*V*_12Gy_), and dose spread (Dspread) were compared between GK SRS and RapidArc SRS for multi-mets. RapidArc plans have smaller CI but larger GI. *V*_12Gy_ are comparable. GK appears better at reducing only very low-dose spread (<3 Gy). The treatment time of RapidArc SRS is significantly reduced compared to GK SRS.

## Introduction

Brain metastases represent the most frequent brain tumor and are a significant cause of morbidity and mortality. Surgery, whole-brain radiation treatment (WBRT), and stereotactic radiosurgery (SRS) are all used in the treatment of brain metastases (1–9). Radiosurgery has emerged as a common treatment modality for brain metastases since the introduction of Gamma Knife (GK) (10), but now advances in technology permits newer techniques, such as Cyberknife^®^ and linear accelerator (LINAC)-based volumetric modulated arc therapy (VMAT) treatment.

Linear accelerator-based systems are capable of achieving delivering treatment techniques common to GK SRS. These include (a) an ensemble of convergent beams or arcs used to target a circumscribed, well-defined lesion, (b) high doses delivered to the planning target volume (PTV), and (c) steep dose gradients created at the margin of the tumor and normal tissue, thus ensuring a low dose outside the target. The common techniques of LINAC SRS include the use of multiple conformal arcs or multiple static intensity modulated beams (IMRT), which further evolved into VMAT (11), to treat a single target positioned at the LINAC isocenter. The treatment time for one target typically ranges from 15 to 20 min, which becomes the limit factor to treat more than 4–5 brain metastases in a single session.

Some studies proposed treating multiple brain metastases SRS with a single virtual isocenter using VMAT technique (12–19), and some compared to GK (16–19). While some previous studies have appeared to show GK as superior to LINAC SRS with regard to normal brain exposure (16–18), a more recent study suggested equivalent low-dose spread and increased delivery efficiency (19) when using non-coplanar RapidArc [one of the VMAT techniques implemented by Varian Eclpise treatment planning system (TPS)]. However, the low-dose spread in this study was defined as 25% of prescription dose (4.5 Gy as the lowest comparison dose), which may not be as low as the dose used by other controversy studies. Therefore, we performed the current study to evaluate the dosimetry and efficiency of these two modalities – GK and LINAC single-isocenter non-coplanar RapidArc SRS for multiple brain metastases, with a special focus on the comparison of very low-dose spread (≤3 Gy). The other difference in our study is that a newer model of GK (Perfexion™) is used as compared to the older model (C/4C) used in literature (19).

## Materials and Methods

### Patients

We used the image data of six patients with three to four brain metastases who received SRS treatment at our institution. The study was approved by our Institutional Review Board (IRB). The cases were planned with GammaPlan^®^, the Gamma Knife Perfexion TPS (Elekta AB, Stockholm Sweden), and Eclipse™ TPS using RapidArc^®^, a particular implementation of VMAT (Varian Medical Systems, Palo Alto, CA, USA) on Varian delivery systems, the more recent versions of which, allow for the delivery of multiple non-coplanar arcs. Each of the GK plans was designed by an experienced GK physicist and approved by an attending neurosurgeon and radiation oncologist. All RapidArc plans were generated by an experienced physicist dedicated to SRS.

### Imaging Protocol

Our treatment planning employed both high-quality magnetic resonance imaging (MRI), from a 1.5-T or 3.0-T scanner, and high-resolution computed tomography (CT) images. The CT technique utilized a 512 × 512-pixel resolution and slice thickness of 1.25 mm to reduce partial volume effects. Contrast enhanced, thin cut (1.0–1.5 mm thickness) 3D T1-weighted MRI was acquired to optimize planning fidelity. Target and normal structure contours were outlined based on the high-resolution MRI and approved by an attending neurosurgeon and radiation oncologist. The contoured MR images were then fused to the CT. The same sets of images and structures were used in both Elekta GammaPlan and Varian Eclipse TPSs.

### Radiation Dose

Stereotactic radiosurgery doses, prescribed according to size of the lesions, varied from 15 to 24 Gy according to RTOG 9508 (20). Radiation doses were modified if unable to meet nearby organ at risk (OAR) tolerance, including optic nerve and chiasm max dose of 10 Gy and brainstem max dose of 12 Gy. Prescription dose and tumor volume of each target are listed in Table 1.

**Table 1:** **Prescription dose and tumor volumes of each target in this study**.

Patient no.	No. of targets	Total tumor vol. (cc)	Tumor vol. (cc)	Prescription dose (Gy)
1	3	5.32	2.53	16
			0.81	24
			1.98	20
2	3	2.08	0.41	24
			0.51	24
			1.16	18
3	3	1.19	0.41	24
			0.52	24
			0.26	24
4	3	5.13	4.62	18
			0.12	24
			0.39	24
5	3	11.14	10.51	15
			0.35	24
			0.28	24
6	4	1.70	0.68	24
			0.56	24
			0.46	24
			0.4	24

### Gamma Knife Treatment Planning

Gamma Knife plans were performed for all patients using the Elekta GammaPlan TPS (version 10.1) for a Gamma Knife Perfexion treatment unit. The Perfexion has 192 Co-60 sources, which are placed on eight sectors. Each position corresponds to a different size collimator. Each sector has 24 sources and three different sizes of open collimators are available for each source (16, 8, and 4 mm) as well as a blocked collimator. Because each of the eight sectors can move independently, it is possible to create plans with composite multiple shots where each sector is of different collimator size. Treatment plan of each lesion starts from automatic shots fill in with composite small to medium sized collimators depending on the volumes of the target, followed by an optimization with inverse planning setting of 99% coverage. After the initial optimization, it usually achieves ~95% coverage with dose distribution that is not very conformal. Planner adjustments are then introduced to achieve >99.5% volume coverage by the prescription dose and a more conformal dose distribution. Adjustment includes changing position and weight of each existing shot and adding new shots. Multiple shots plans were usually used to increase conformity, rather than fewer shots to minimize treatment time. All targets are prescribed to 50% isodose line. The “TMR 10” dose algorithm is used in Gamma plan calculation. Detailed GK planning parameters are listed in Table 2.

**Table 2:** **Planning parameters for Gamma Knife Perfexion plans**.

Patient no.	No. of targets	Number of shots	Beam-on time (min)	Est. total tx time (min)
1	3	43	83.5	100
2	3	15	72.2	87
3	3	4	47	56
4	3	19	70	84
5	3	26	93	112
6	4	10	64	77

### RapidArc Treatment Planning

For all patients in the study, RapidArc plans were optimized using four to six non-coplanar partial arcs (21). Collimator and couch angles as well as single arc lengths were optimized for each individual case with small adjustments to our established template. The detailed planning parameters for each case are listed in Table 3. Inverse planning was performed with the Varian Eclipse TPS (version 11) and dose calculation with a grid resolution of 1.0 mm. Three layers of tuning rinds, as described by Clark et al. (14), were constructed to control the dose–volume constraints corresponding to the high-, mid-, and low-dose levels, where “high” is the prescription dose of each target, “medium” the 12-Gy dose level, and “low” the 6-Gy dose level. The outer diameters of each layer of rinds depend on the size of the target. All plans were constructed using the 6-MV flatten filter free (FFF) beam, generated by a TrueBeam STx radiosurgery system equipped with a high-definition (2.5 mm) multileaf collimator (MLC). The High Intensity Mode unflattened 6 MV beam delivers radiation at a dose rate of 1400 cGy/min. Prendergast first showed the efficiency benefit of unflattened beams for cranial treatments (22). The HD120 MLC™ has 120 leaves with a leaf width projected at isocenter of 2.5 mm for the central 8.0-cm region and 5.0 mm for the two 7.0-cm peripheral regions (23, 24). One isocenter was used for all targets and was placed at the center of mass of all targets determined by the TPS. Each lesion must have >99.5% volume covered by prescription dose. “AAA” algorithm with heterogeneous correction, 1 mm calculation grid is used for final dose calculation.

**Table 3:** **Planning parameters for single-isocenter VMAT plan**.

Patient no.	No. of targets	No. of arcs	Table angles (Varian IEC Scale)	Arc length	Monitor units (MU)	Beam-on time (min)	Est. total tx time (min)
1	3	4	45, 0, 335, 300	170, 120, 140, 140	7000	5.0	15
2	3	6	70, 40, 10, 0, 335, 305	160, 150, 40, 130, 160, 150	8300	5.9	18
3	3	6	50, 25, 0, 340, 315, 290	160, 160, 130, 140, 140, 150	9600	6.9	21
4	3	5	80, 45, 20, 353, 300	140, 140, 150, 140, 150	10,130	7.2	22
5	3	5	60, 20, 345, 310, 270	120, 120, 120, 140, 140	8660	6.2	19
6	4	5	70, 40, 10, 0, 300	170, 170, 170, 140, 140	9750	7.0	21

### Evaluation Tools

Dosimetric parameters for analysis included RTOG conformity index (CI), gradient index (GI), 12 Gy isodose volume (*V*_12Gy_) for each target, and dose spread (Dspread) for each plan. RTOG CI = PV/TV, where PV is the prescription volume and TV is the target volume (25). Paddick GI = PV50%/PV, where PV50% is 50% of the prescription isodose volume and PV is the prescription volume (26). Dspread reflects the SRS plan’s capability of confining radiation to within the lesions’ local vicinity minimizing spread to surrounding normal brain tissues. Each plan has a dose (Dspread) on the total tissue DVH, such that once dose drops below Dspread, the isodose volume starts increasing dramatically. In this study, Dspread is defined at the dose at which the volume increase first exceeds 20 cc/0.1 Gy drop in dose. For the Eclipse TPS, it is the dose at 2 cc/cGy on a differential DVH curve for normal brain tissue. Treatment times for RapidArc SRS were estimated using the summation of patient setup time, image guidance and verification (IGRT) time, and radiation delivery time. Radiation delivery is based on the total dose divided by 1400 cGy/min dose rate. Treatment time for GK SRS was estimated using the summation of patient setup time, shot transition time, and net beam-on time. Beam-on time assumes new Co-60 sources whose dose rate is 360 cGy/min.

In order to compare the results across different TPSs, the three-dimensional (3D) radiation dose matrix of both GK and RapidArc SRS plans were exported in DICOM RT format to a third party system (MIMVISTA). Both dose matrices encompass the entire brain at a dose calculation resolution of 1.0 mm.

### Statistics

Prism^®^ version 5.0 was used to process data and perform statistical analyses. Direct comparison was performed *via* paired Wilcoxon signed rank test; multivariate regression was performed *via* least squares regression with an identity link function. *p*-values <0.05 were considered significant.

## Results

**Figure 1:**
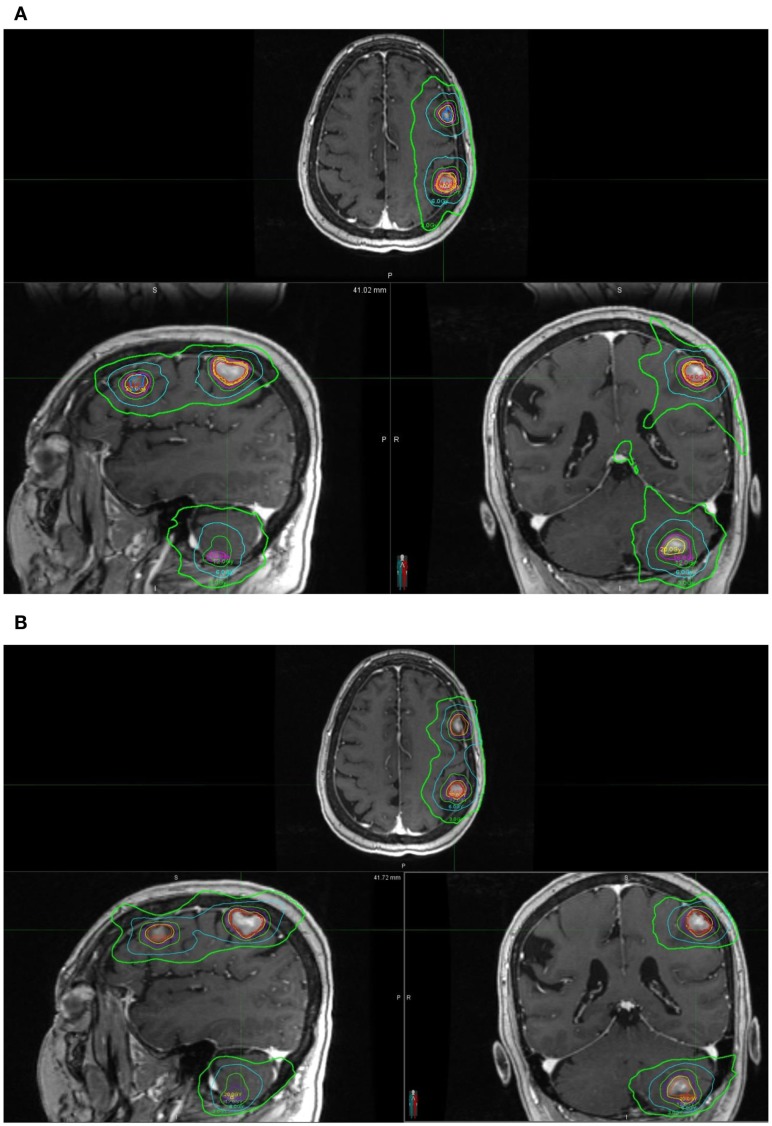
**Isodose distribution of a single-isocenter VMAT (RapidArc) SRS plan (A) and Gamma Knife (B) Perfexion plan for the same patient (patient #1)**. Isodose lines are 24, 20, 16, 12, 6, and 3 Gy. **(A)** Isodose distribution of a single-isocenter VMAT (RapidArc) SRS plan for patient #1. **(B)** Isodose distribution of Gamma Knife Perfexion SRS plan for patient #1.

**Figure 2:**
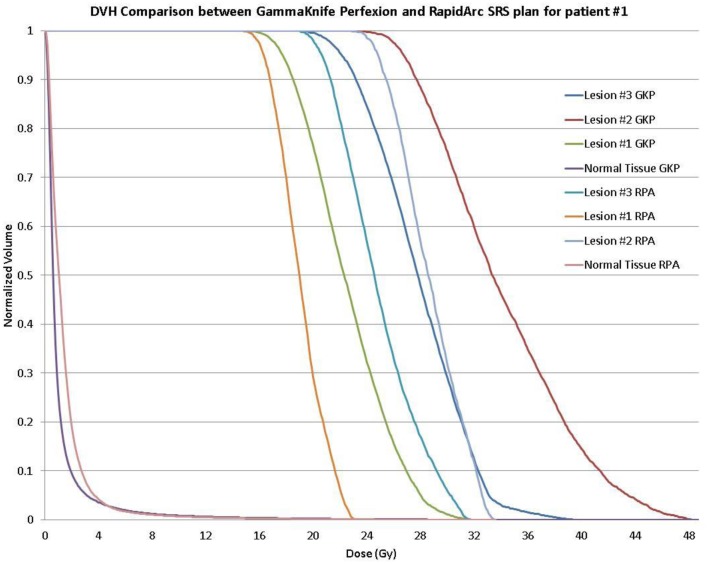
**Dose–volume histogram (DVH) comparison of a single-isocenter VMAT (RapidArc) SRS plan and Gamma Knife Perfexion plan for the same patient (patient #1) showing background dose to normal tissue and DVH for the 16, 20, and 24 Gy treatment of the larger to the smaller target, respectively**.

Clinical acceptable plans were achieved by both GK SRS and RapidArc SRS. Figure 1 shows the isodose distribution of both GK plan and RapidArc plan for patient #1, a typical patient. Figure 2 shows the dose–volume histogram of three lesions and normal brain tissues of both plans for this patient.

RapidArc SRS has smaller CI (1.19 ± 0.14 vs. 1.50 ± 0.16, *p* < 0.001), however, a larger GI (4.77 ± 1.49 vs. 3.65 ± 0.98, *p* < 0.01). The larger GI values for the RapidArc SRS plan are not because they have larger 50% prescription isodose volume but because they all have smaller 100% prescription isodose volume. Therefore, instead of comparing GI values, which are not a valid comparison, the absolute volume that receives more than 12 Gy (*V*_12Gy_) is compared in our study. *V*_12Gy_ was chosen because it is a known predictor of radiation toxicity in normal brain tissues (27). The *V*_12Gy_ of each individual targets were comparable between GK and RapidArc, 3.06 ± 2.20 vs. 2.73 ± 1.38 cc, respectively, *p* = 0.58. Table 4 shows the CI, GI, and *V*_12Gy_ of each individual targets. To further evaluate the impact on the dose to normal brain tissue outside the target, we compared multiple dosimetric parameters for low-dose spread. Lower dose spread V6, V4.5, and V3 were also equivalent (Table 5).

**Table 4:** **Comparison of conformity index and normal tissue *V*_12Gy_ of each target in this study**.

							Normal tissue	
Patient #	Target #	Target vol. (cc)	Conformity index		Gradient index		*V*_12Gy_ (cc)	
			GKP	RPA	GKP	PRA	GKP	RPA
1	1	2.53	1.62	1.15	3.42	4.28	4.80	3.45
	2	0.81	1.63	1.17	3.75	4.63	4.14	3.59
	3	1.98	1.60	1.14	3.21	3.92	5.76	4.51
2	4	0.41	1.27	1.12	3.46	5.04	1.39	1.91
	5	0.51	1.43	1.14	2.82	4.55	1.55	2.13
	6	1.16	1.78	1.38	2.75	4.54	2.72	3.21
3	7	0.41	1.54	1.15	2.56	4.68	1.20	1.91
	8	0.52	1.35	1.10	3.71	4.25	2.08	2.19
	9	0.26	1.58	1.23	3.88	5.34	1.33	1.54
4	10	4.62	1.29	1.15	3.64	3.69	7.23	6.48
	11	0.12	1.83	1.67	3.32	10.30	0.61	1.94
	12	0.39	1.51	1.26	2.76	5.14	1.24	2.13
5	13	10.51	1.36	1.07	2.69	2.58	8.64	5.34
	14	0.35	1.49	1.26	4.60	5.20	2.04	1.94
	15	0.28	1.68	1.21	6.40	5.38	2.73	1.55
6	16	0.68	1.41	1.09	4.38	4.07	3.52	2.33
	17	0.56	1.46	1.11	4.95	4.55	3.50	2.26
	18	0.46	1.41	1.17	4.45	4.19	2.43	1.80
	19	0.4	1.37	1.00	2.67	4.30	1.28	1.58
Mean			1.50	1.19	3.65	4.77	3.06	2.73
SD			0.16	0.14	0.98	1.49	2.20	1.38
			*p*	<0.001	*p*	0.01	*p*	0.58

**Table 5:** **Dosimetric parameters of Gamma Knife SRS and RapidArc SRS radiation treatment plans in this study**.

	Gamma Knife		RapidArc		
	Mean	Std.	Mean	Std.	*p*
V12 (patient composite)	10.85	7.2	9.7	5.1	0.63
V6	36.9	16.9	36.3	14.7	0.96
V4.5	86.7	29.8	99	27.3	0.15
V3	160.8	55.7	224	53	0.1
D100	4	0.9	4.6	0.6	0.3
D200	2.6	0.6	3.2	0.5	0.06
D300	2	0.4	2.6	0.4	<0.05
Dspread	1.9	0.73	2.5	0.2	0.01
Beam-on time	71.6	15.9	6.4	0.8	<0.01
Est. total tx time	85.9	19.1	19.3	2.6	<0.01

To further evaluate the low-dose spread between the two techniques, we also evaluated the dose to 100, 200, and 300 cc normal brain tissues (Table 5). The dose to 100 cc (D_100cc_) is equivalent. The dose to 200 cc (*D*_200cc_) is approaching a statistically significant difference and favors GK plans, 2.6 vs. 3.2 Gy, *p* = 0.06. The dose to 300 cc normal brain tissue (D_300cc_) is better for GK plans, 2 vs. 2.6 Gy, *p* < 0.05. However, the very small absolute difference of 0.6 Gy is not considered clinically significant in either case.

In addition, we also used the parameter of Dspread, which is defined at the dose when volume increase exceeds 20 cc/0.1 Gy dose decrease, for easy comparison of low-dose spread between plans. GK plan had smaller Dspread, 1.9 vs. 2.5 Gy, *p* = 0.01. Again, the small difference at this very low-dose level is not expected to be clinically significant.

We also evaluated the treatment delivery time for both GK and RapidArc SRS plans. For these six cases, beam-on time averages ~72 min for GK SRS and 6.4 min for RapidArc SRS (Table 5). Total treatment time for GK is estimated between 56 and 112 min including setup and shot transition time, based on new radiation sources. Total treatment time for RapidArc SRS is estimated between 15 and 22 min including setup, imaging guidance, and treatment table rotation time between arcs. It is average ~4.5 times shorter compared to GK treatment (Tables 2 and 3).

## Discussion

Since the invention of GK in the 1950s, multiple C-arm LINAC treatment machines have been commonly used for cranial radiosurgery. C-arm LINAC treatment machines have the advantage of being versatile, capable of delivering treatment to extracranial sites as well as intracranial sites and capable of delivering multi-fraction treatment with easily reproducible non-invasive target immobilization and localization. With the availability of image-guided radiation therapy, advances in computer science, and improvement of treatment delivery hardware, such as high-definition MLCs, which allow for the simultaneous delivery of shaped dose to multiple targets, modern LINACs are able to achieve the same degree of accuracy and precision of the GK but with more treatment efficiency. With the more recent development of VMAT optimization planning capability, LINACs are able to simultaneously treat multiple targets with high plan quality and even greater efficiency. This becomes particular appealing with SRS for multiple brain metastases. In our current study, we have demonstrated, along with others (19), that RapidArc-based treatment planning can achieve the same target coverage as GK, with similar clinically acceptable dosimetry results (Table 4). Even with multiple-shot optimized plans, the CI of GK plans are still inferior to that of RapidArc plans. The major concern for the RapidArc plans is the very low-dose spread to the normal brain tissue outside the treatment fields. Several other studies have evaluated this and had slightly different conclusions. We paid special attention to the low-dose spread in the current study. With our analysis, the statistical difference in low-dose spread is only at a very low-dose level, particularly <3 Gy. This small amount of very low-dose spread is not considered clinically significant and is comparable to the dose of one fraction of whole-brain irradiation. Extensive evidence have demonstrated that this is below the normal tissue tolerances of all sensitive brain structures, such as cochlea, optic nerve, chiasm, brainstem, and hippocampus (28–32). A recent assessment of the true risk of very low doses to normal brain tissues is highlighted in a recent study from the University of Florida (33). They analyzed 23 years of LINAC-based SRS data to address the long-term malignancy risk of low doses to normal brain compared to epidemiological brain tumor data in Florida. An analysis of the 627 cranial SRS patients (out of a total cohort of 2369 analyzed), who had five or more years of follow-up, showed that there was no increased risk of malignancy compared to the general population.

One of the most significant advantages of RapidArc compared to GK is the efficiency of treatment delivery. In our current study, we included patients with up to four brain metastases, based on level-1 evidence (5–7). For RapidArc SRS, beam-on time is <10 min for all the patients. Including setup and image-guidance procedures, the total treatment time can easily be <15–20 min. This efficiency benefit is primarily due to three key features of modern LINAC technology in this study: (1) single-isocenter–non-coplanar VMAT (RapidArc SRS) treatment for all the targets (14, 19) vs. multiple isocenters corresponding to each individual target; (2) high-resolution MLC (HD120 MLC) – providing simultaneous high-resolution beam shaping (23, 24) of all the targets simultaneously from any angle vs. one target at a time; and (3) high dose rate (22) provided by the High Intensity Mode vs. the maximum dose rate of Co-60, which declines by 50% of max over 5 years. This not only increases the machine utilization but also improves the patient experience, particularly with a frameless immobilization system. The delivery efficiency of GK can be improved by using a large helmet size and less complex plans with fewer shots. However, this will result in a lower conformity index and increases low-dose spread. Recent high-level evidence from Japan suggests that SRS alone is safe and appropriate to consider for patients with up to 10 brain metastases (34). The advantage of delivery efficiency of RapidArc would be even greater.

Compared to other studies using single-isocenter coplanar VMAT (15, 16, 18) techniques, this study used a single-isocenter, multiple arc technique, with multiple treatment angles. And compared to previous published studies using RapidArc SRS single-isocenter multi arc VMAT techniques (13, 14, 19), the arc geometry utilized in the current study is different. Multiple (4–6) non-coplanar partial arcs, between 110° and 150° arc lengths, were used, which avoids a transverse plane full 360° arc. The rationale is that for the transverse plane full 360° arc, since the second half arc enters through the exit of the first half arc, the normal brain tissue will receive more doses. By offsetting the arcs from the transverse plane, parallel-opposed beams will be avoided and the normal tissue isodose volume can be reduced, as Schell et al.’s study showed previously (21).

The target volumes in this study ranged from 0.1 to 10.5 cc, with most of them <2 cc (small targets). A recent planning comparison study for large target fractionated SRT using VMAT and GK for brain metastases and gliomas showed that GK produce better dose distribution for target volumes below 15 cc, while VMAT results in better dose conformity to the target and lower doses to the OARs for larger or irregular volumes (35).

It is worthwhile to point out that while the prescription dose covering the target volume is the same between GammaKnife and RaridArc SRS plans, the dose heterogeneity within the planning target volume (PTV) is much different. GK plan for each target in this study is prescribed to the standard 50% isodose line, which implies that the maximum dose within the PTV is twice as high as the prescription dose. RapidArc plan is an inverse planning process; the optimization goal to each PTV set by the planner is to have at least 99.5% volume receiving prescription dose. No constraint is set on the maximum dose. Therefore, there is no manual selection of a specific normalization isodose line for RapidArc plans. The ratio of prescription dose to maximum dose for the 19 targets in RapidArc plans is 63 ± 5% (range 54–74%).

## Conclusion

Dosimetric parameters reflecting prescription dose conformality (CI), dose fall off (GI), radiation necrosis indicator (*V*_12Gy_), and dose spread (Dspread) were compared between GK and RapidArc for multi-mets SRS for a cohort of six patients with up to four metastases. RapidArc SRS plans have smaller CI but larger GI. *V*_12Gy_ are comparable. GK SRS performs better at reducing very low-dose spread. However, the lowest doses to normal tissues in properly optimized RapidArc plans are not considered clinically significant (<3 Gy). While several prior studies demonstrated the clinical feasibility of delivering high quality plans for single-isocenter RapidArc SRS for multiple cranial metastases, they did not address the question of very low dose to normal brain. This study quantified the low-dose spread in the case of three or four metastases. It also demonstrated that low dose is not clinically significant, even though it is larger than that of GK. On the other hand, the treatment time of RapidArc SRS is significantly reduced compared to GK SRS. Treatment time for SRS to multiple cranial metastases will become increasingly more relevant with the trend toward treating greater numbers of brain metastases in a single SRS session and as systemic therapies targeting the primary cancer succeed in extending survival in this increasing population of patients.

## Ethics

The study involved human subjects: Y. The study was approved by IRB of Thomas Jefferson University.

## Author Contributions

All authors involved in the design and data collection. HL wrote the manuscript. All authors reviewed and edited the manuscript. All authors have read and approved the final version of this manuscript, all believe that the information presented is true and correct, and all are willing to take public responsibility for the manuscript.

## Conflict of Interest Statement

Haisong Liu, Ph.D. and Wenyin Shi, M.D., Ph.D. have consulting agreements with Varian Medical Systems, Inc., Palo Alto, CA, USA. The remaining authors declare that the research was conducted in the absence of any commercial or financial relationships that could be construed as a potential conflict of interest.
